# Case Report: Transcatheter closure of a giant post-traumatic femoral arteriovenous fistula using ventricular septal occluder

**DOI:** 10.3389/fsurg.2023.1295595

**Published:** 2024-01-04

**Authors:** Zewei Chen, Yiyuan Huang, Chenxi Ye, Jiale Liu, Yuxin Liu, Yirui Tang, Zhenfei Fang

**Affiliations:** ^1^ Department of Cardiovascular Medicine, the Second Xiangya Hospital, Central South University, Changsha, Hunan, China; ^2^ Xiamen Cardiovascular Hospital, School of Medicine, Xiamen University, Xiamen, Fujian, China

**Keywords:** arteriovenous fistula (AVF), vascular injury, traumatic arteriovenous fistula, vascular interventional therapy, ventricular septal occluder

## Abstract

Giant femoral arteriovenous fistulas are comparatively uncommon, typically treated through covered stents, coil embolization, and open surgical repair. Nevertheless, these options may not be appropriate for all patients. Herein, we describe a case of traumatic femoral arteriovenous fistulas that led to drastic dilatation of the femoral arteriovenous system and considerable heart failure symptoms due to prolonged lack of treatment. Given the intricate anatomical location of the fistula and the patient's severe cardiac dysfunction, surgical repair is often unfeasible. Consequently, we adopted an innovative approach in this case, utilizing a ventricular septal occluder device for fistula closure. This constitutes the first report of an arteriovenous fistula transcatheter closure with a septal occluder.

## Introduction

Arteriovenous fistulas (AVFs) are abnormal connections between arteries and veins, and it can be divided into congenital or acquired depending on its etiology. Congenital AVFs occur due to abnormal capillary development, which makes arteries and veins connect directly without intermediate capillaries. Acquired AVFs can be further subdivided into surgically created, or secondary to iatrogenic injury or trauma. Therapeutic alternatives for managing AVFs comprise ultrasound-guided compression, stent placement, coil embolization, or surgical repair (1, 2). Early intervention becomes crucial for large AVFs as they pose a heightened risk for adverse complications including thrombosis and heart failure due to their reluctance to close spontaneously (3). In the presented case, a large AVF remained untreated for approximately 50 years, leading to severe heart failure, thereby complicating surgical repair. A transcatheter procedure was thus employed, utilizing a ventricular septal defect (VSD) occluder, to successfully close the AVFs, substantially improving the patient's symptoms.

## Case report

Approximately a decade prior to the current assessment, a female patient aged 59 was first admitted to our hospital presenting with symptoms of shortness of breath and chest tightness. Her clinical laboratory test results revealed a N-terminal prohormone BNP (NT-proBNP) level of 3,556 pg/ml, significantly above the normal range of 0–125 pg/ml. Transthoracic echocardiography (TTE) depicted an enlargement of the heart (with LVDd 65 mm, LAS 53 mm, RVD 47 mm, RAS 47 mm), a patent ductus arteriosus (PDA), and a normal ejection fraction indicative of healthy left heart function, at 66%. Believing PDA to be the potential cause of the patient's heart failure symptoms, we decided to proceed toward its occlusion. In the course of the surgery, a palpable thrill was identified under the right inguinal ligament. A review of the patient's medical history revealed a traumatic incident involving a fire spear at the age of seven, which caused an injury in the right groin. This palpable thrill could potentially be attributed to the development of an arteriovenous fistula, to which the patient had previously declined treatment. Consequently, successful occlusion of the PDA was carried out via puncturing the left femoral artery. Following the surgical procedure, we observed a gradual improvement in the patient's initial symptoms, with a noted decrease in the levels of NT-proBNP to 1,115 pg/ml—significantly lower than the pre-surgery levels. Although we suggested consulting with the Vascular Surgery Unit to treat the femoral arteriovenous fistula, the patient didn't heed our advice. Post-surgery, the patient appeared to perform physical activities without difficulty. Upon discharge, we assessed her heart function as New York Heart Association class I.

**Figure 1:**
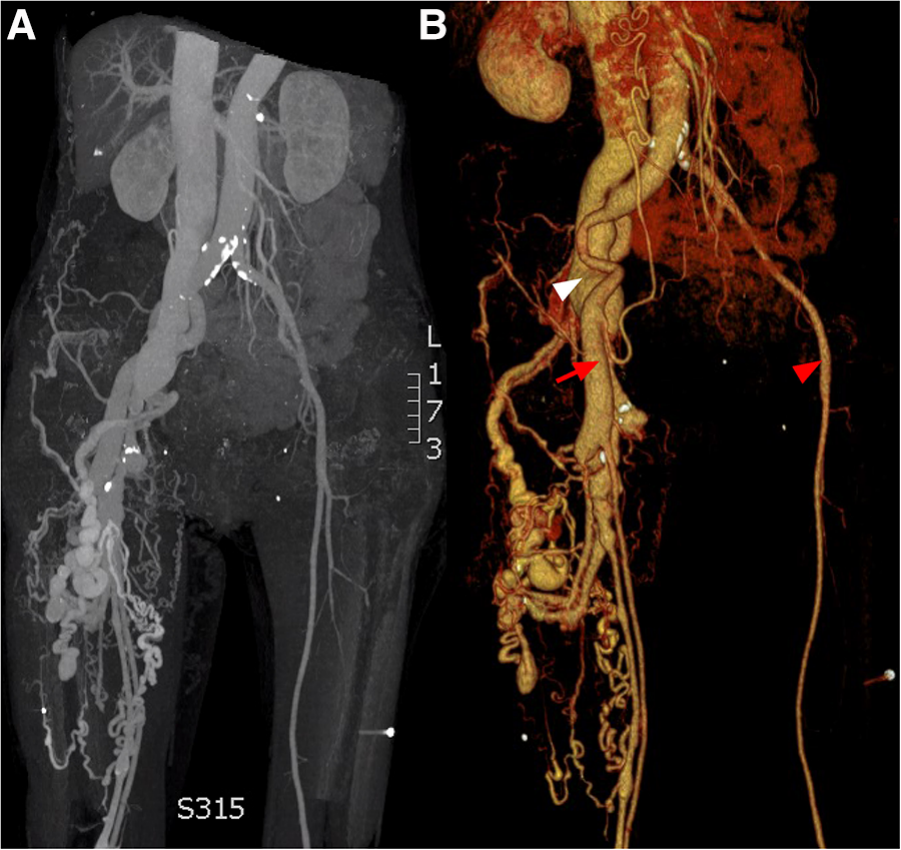
CT angiogprahy: (**A**) coronal section, (**B**) 3D reconstruction. Normal femoral artery (red arrow head), dilated femoral artery (red arrow) and aneurysmal dilatation of the iliac vein (white arrow head).

**Figure 2:**
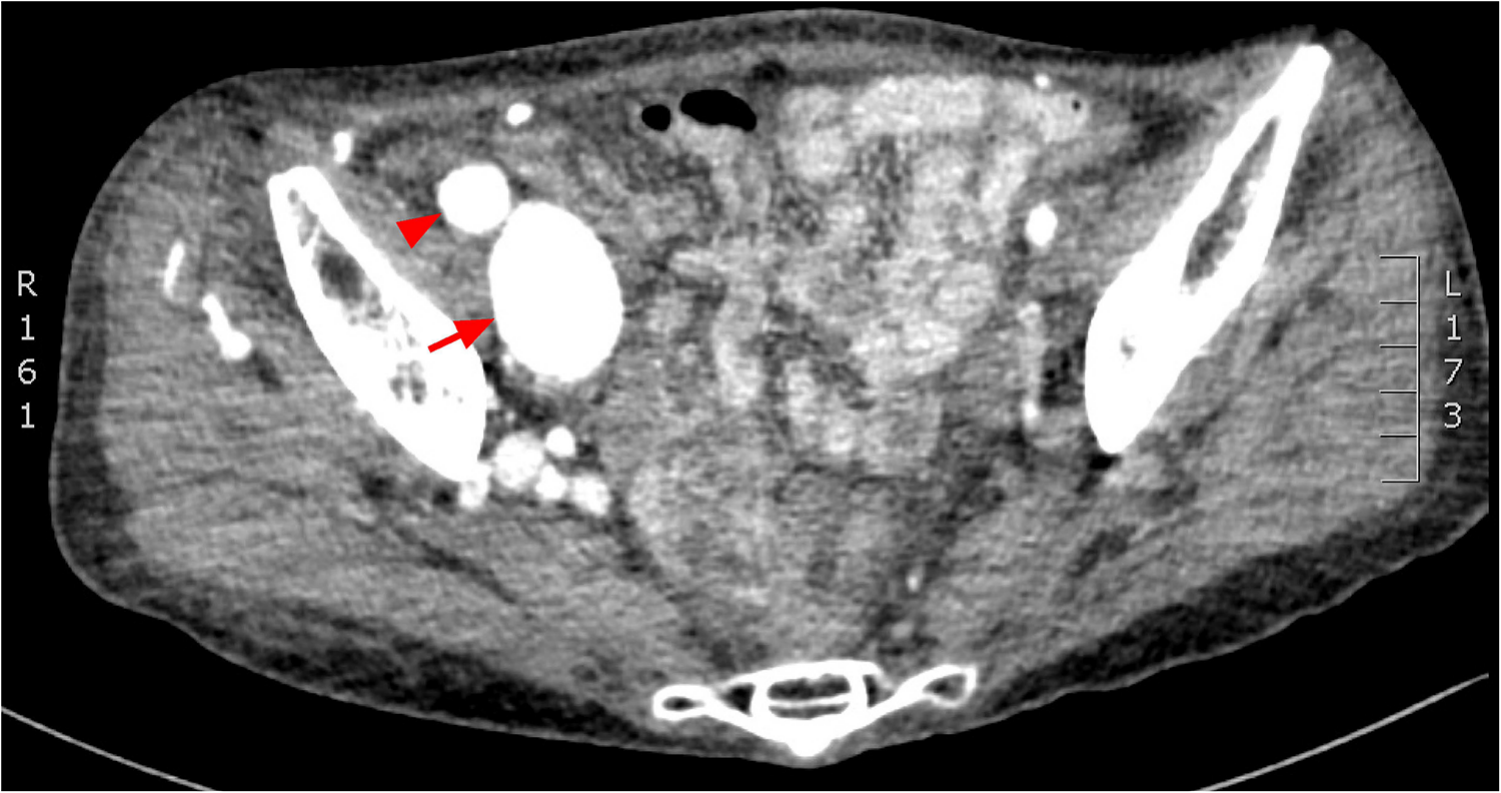
CT angiogprahy: axial section, dilated common iliac artery (arrow head) and vein (arrow).

**Figure 3:**
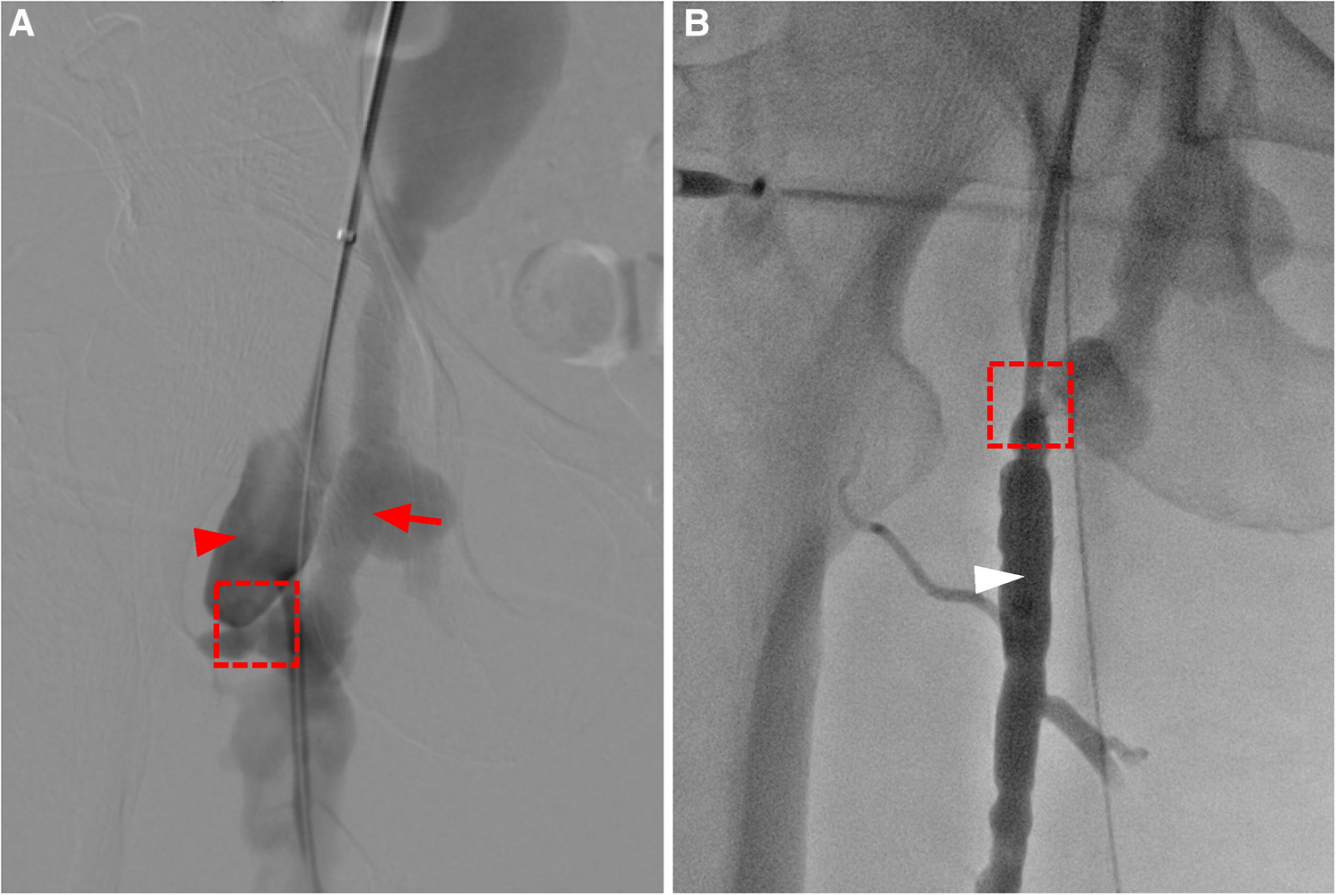
(**A**) angiography shows enlargement of the femoral artery (red arrow head), and a fistula connecting (dashed box) the deep femoral artery and the femoral vein, leading to early enhancement and aneurysmal dilatation of the femoral vein (red arrow). (**B**) The catheter enters the femoral vein (white arrow head) through the fistula through the deep femoral artery.

**Figure 4:**
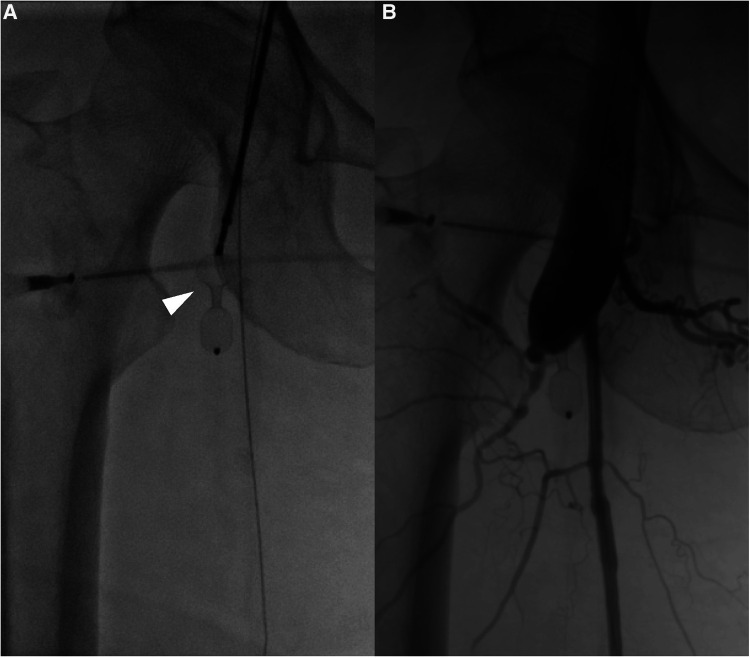
(**A**) An interventricular septal occluder is positioned at the fistula between the deep femoral artery and femoral vein (white arrow head). After the placement of the occluder, angiography shows that the fistula was closed with only a small residual shunt.

Ten years post-PDA occlusion, the patient was readmitted to our hospital due to a year-long exacerbation of breathlessness. The physical examination revealed swelling and elevated skin temperature in the right leg, along with an audible murmur in the right inguinal region. Laboratory tests indicated an N-terminal prohormone BNP level of 9,120 pg/ml (reference: 0–125 pg/ml) and a high-sensitivity troponin T level of 10 pg/ml (reference range: 0–100 pg/ml), while blood routine, liver function, renal function, and electrolyte tests were normal. TTE illustrated expanded heart chambers (LVDd 88 mm, LAS 53 mm, RVD 39 mm, RAS 48 mm), diffused ventricular wall motion weakness, and reduced left heart function (ejection fraction: 30%). This suggested a 36% reduction in left ventricular ejection fraction and progressive heart dilation compared to the state a decade ago, indicating deteriorating heart failure. An ultrasound of his lower extremity vasculature identified a 3.9 mm fistula between the right deep femoral artery and the right femoral vein with high-velocity color flow. Subsequently, a CT angiography scan revealed severe dilation from the inferior vena cava to the right femoral vein and aneurysms in the dilated right common iliac artery, internal iliac artery, and external iliac arteries (Figures 1, 2). Given these results, open surgical repair was deemed unfeasible due to heart function deterioration. The deployment of a covered stent was dismissed as overly risky given it would obstruct numerous deep femoral artery branches. The patient's blood vessels are severely dilated and distorted, further complicating matters, as it renders the stents unfixable. Instead, a novel approach to fistula closure using ventricular septal occlude was ventured under local anesthesia with fluoroscopic and ultrasound guidance. The left femoral artery was percutaneously accessed under vascular ultrasound guidance. Intravenous heparin (50 IU/kg) and antibiotic prophylaxis were administered. A 4-Fr angiographic catheter (VER135°, Cordis, Corporation, Miami, FL, USA) was introduced into the abdominal aorta and right common iliac artery with a noodle wire (AGA Medical, Golden Valley, MN, USA), detecting the AVF. Angiographies performed with the VER135° catheter visibly showed the abdominal aorta, dilated right iliac artery, and early enhancement of the inferior vena cava. Femoral AVF was confirmed on angiogram at the beginning of the deep femoral artery with an large aneurysm connecting with the common femoral vein (Figure 3). Thereafter, the VER135° catheter was introduced into the deep femoral vein across the AVF with a 260-cm long noodle wire (AGA Medical, Golden Valley, MN, USA). The left femoral vein was percutaneously accessed, and the noodle wire was snared and pulled out with an Amplatz gooseneck snare (Microvena, White Bear Lake, MN, USA) introduced from the femoral vein, establishing a stable arterial-venous wire loop through the fistula. A Lifetech delivery sheath was introduced over the wire from the femoral vein through the AVF to the femoral artery. Under fluoroscopic guidance, an 8 mm (3–5 mm larger than the size of the fistula) VSD occluder (Lifetech, HeartR, XJFVJ08) with its corresponding delivery cable was inserted through the sheath and the device was deployed in the AVF. Aortography was done again to confirm fistula closure, with a small residual shunt remaining (Figure 4). On the first postoperative day, the patient exhibited reduced respiratory distress, the pulsation of the right dorsalis pedis artery normalized, and the ankle brachial index (ABI) measured 1.29 (compared to a preoperative ABI of 0). Treatment included oral anticoagulants, and by the third postoperative day, the patient was discharged in a stable clinical condition.

## Discussion

Traumatic femoral arteriovenous fistulas (AVFs), although relatively rare, can result from gunshot wounds, stab wounds, and iatrogenic injuries (1, 4, 5). While any part of the body can develop a traumatic AVF, extremity vessels are most commonly affected (4, 6). It's pertinent to note that iatrogenic injuries are becoming the primary cause of femoral AVF due to the significant rise in cardiac catheterizations. Research suggests that approximately 1% of cardiac catheterization patients develop a femoral AVF (7, 8).

The manifestation of arteriovenous fistulas, abnormal connections between the vein and artery, varies based on the duration of the ailment. Initial symptoms may only show positive signs during physical examinations, including a machinery murmur, palpable thrill, or pulsatile mass at the focus (4). Over time, some patients may exhibit elevated skin temperature. If high-flow AVFs prolong along with the disease duration, the end result could be high-output cardiac failure (3, 4, 9). Studies show that between 1.4% and 13% of patients with traumatic AVF experience heart failure, with every six-year delay in AVF presentation post-injury increasing heart failure risk by 30% (1, 5). Asymptomatic AVF patients are also at risk of thrombosis (10). For example, our case study patient was diagnosed 52 years post-initial trauma, presenting primary symptoms such as lower limb swelling, shortness of breath, and chest tightness. The prolonged delay in treatment resulted in heart overload, ultimately leading to heart enlargement and failure.

AVF diagnosis primarily depends on imaging examinations like color Doppler ultrasound, CT angiography, MR angiography, and digital subtraction angiography (DSA) (11). DSA, typically considered the gold standard approach for diagnosing AVF, can accurately showcase the fistula's location, size, and relation with adjacent blood vessels. Nevertheless, the invasive nature of DSA could pose potential risks, including the possibilities of hemangioma rupture or thrombus detachment. Although DSA is the most definite diagnostic tool available, studies suggest that CT angiography may have a superior initial diagnostic utility, with sensitivity and specificity ranges of 90%–100% and 98%–100% respectively for detecting vascular lesions (12, 13).

In general, AVF treatments span non-surgical and surgical therapies. Non-surgical treatment largely includes ultrasound-guided compression, introduced by Fellmeth et al. (14), This approach is typically effective for small arteriovenous fistulas with a short course of disease, such as certain iatrogenic femoral arteriovenous fistulas, which do not significantly influence hemodynamics and can often self-heal or be healed through ultrasound-guided compression (7, 15, 16). Research indicates that one-third of iatrogenic AVF close spontaneously within one year, which justifies conservative management for at least this duration (7). However, traumatic arteriovenous fistulas (AVFs) may comprise large fistulas or have a long-standing injury that are not suitable for conservative treatment (14) necessitating active surgical intervention to avert potential complications. Techniques range from covered stents, coil embolization, to open surgical repair of blood vessels (2, 17). Unfortunately, none of these treatments were optimal in this case due to the patient's decreased heart function and heightened risk from open surgery. The issue is further complicated by the patient's severely dilated and distorted blood vessels, making the stents unfixable. Consequently, a ventricular septal occluder was used to seal the fistula under local anesthesia, successfully closing the main fistula—a novel approach for femoral AVF. The majority of previous femoral AVF treatment reports focused on surgical intervention (4). Yet, there have been isolated case reports regarding the use of a septal occluder in assisted AVF treatment (18). As far as we are aware, this is the first instance of a ventricular septal occluder being employed to treat a femoral AVF, thereby providing a new potential avenue for intervention.

## Conclusion

Untreated large arteriovenous fistulas (AVFs) potentially result in various complications over time, including heart enlargement and failure. Therefore, quick medical intervention is necessary upon detection to avert complications associated with delayed treatment. In this case, a severe femoral AVF resulting from post-trauma treatment delay. Our unique treatment strategy involved using a ventricular septal occluder to close the fistula, tailored to the patient's circumstances. This opens up potential alternative treatment possibilities for AVFs.

## Data Availability

The original contributions presented in the study are included in the article/Supplementary Material, further inquiries can be directed to the corresponding author.

## References

[1] Wenzl FA Miljkovic SS Dabestani PJ Kessler JJ 2nd Kotaru TR Kalamchi LD A systematic review and individual patient data meta-analysis of heart failure as a rare complication of traumatic arteriovenous fistulas. J Vasc Surg. (2021) 73(3):1087–94.e8. 10.1016/j.jvs.2020.08.138 33002586

[2] Jayroe H Foley K. Arteriovenous fistula. Treasure Island, FL: StatPearls (2023). 32644639

[3] Huang W Villavicencio JL Rich NM. Delayed treatment and late complications of a traumatic arteriovenous fistula. J Vasc Surg. (2005) 41(4):715–7. 10.1016/j.jvs.2005.01.049 15874939

[4] Asensio JA Dabestani PJ Miljkovic SS Wenzl FA Kessler JJ 2nd Kalamchi LD Traumatic penetrating arteriovenous fistulas: a collective review. Eur J Trauma Emerg Surg. (2022) 48(2):775–89. 10.1007/s00068-020-01574-z 33386864

[5] Robbs JV Carrim AA Kadwa AM Mars M. Traumatic arteriovenous fistula: experience with 202 patients. Br J Surg. (1994) 81(9):1296–9. 10.1002/bjs.1800810912 7953391

[6] Diamond S Gaspard D Katz S. Vascular injuries to the extremities in a suburban trauma center. Am Surg. (2003) 69(10):848–51. 10.1177/000313480306901006 14570361

[7] Kelm M Perings SM Jax T Lauer T Schoebel FC Heintzen MP Incidence and clinical outcome of iatrogenic femoral arteriovenous fistulas: implications for risk stratification and treatment. J Am Coll Cardiol. (2002) 40(2):291–7. 10.1016/S0735-1097(02)01966-6 12106934

[8] Perings SM Kelm M Jax T Strauer BE. A prospective study on incidence and risk factors of arteriovenous fistulae following transfemoral cardiac catheterization. Int J Cardiol. (2003) 88(2–3):223–8. 10.1016/S0167-5273(02)00400-X 12714202

[9] Cakmak M Cakmak N Arikan E Sert A Say AE Ersek B. Congestive heart failure due to traumatic arteriovenous fistula–two case reports. Angiology. (2003) 54(5):625–9. 10.1177/000331970305400515 14565641

[10] Gillespie DL Villavicencio JL Gallagher C Chang A Hamelink JK Fiala LA Presentation and management of venous aneurysms. J Vasc Surg. (1997) 26(5):845–52. 10.1016/S0741-5214(97)70099-5 9372824

[11] Chen JK Johnson PT Fishman EK. Diagnosis of clinically unsuspected posttraumatic arteriovenous fistulas of the pelvis using CT angiography. AJR Am J Roentgenol. (2007) 188(3):W269–73. 10.2214/AJR.05.1230 17312034

[12] Soto JA Munera F Morales C Lopera JE Holguin D Guarin O Focal arterial injuries of the proximal extremities: helical CT arteriography as the initial method of diagnosis. Radiology. (2001) 218(1):188–94. 10.1148/radiology.218.1.r01ja13188 11152800

[13] Soto JA Munera F Cardoso N Guarin O Medina S. Diagnostic performance of helical CT angiography in trauma to large arteries of the extremities. J Comput Assist Tomogr. (1999) 23(2):188–96. 10.1097/00004728-199903000-00005 10096324

[14] Fellmeth BD Roberts AC Bookstein JJ Freischlag JA Forsythe JR Buckner NK Postangiographic femoral artery injuries: nonsurgical repair with US-guided compression. Radiology. (1991) 178(3):671–5. 10.1148/radiology.178.3.1994400 1994400

[15] Mccann RL Schwartz LB Pieper KS. Vascular complications of cardiac catheterization. J Vasc Surg. (1991) 14(3):375–81. 10.1016/0741-5214(91)90090-H 1880845

[16] Zhou T Liu ZJ Zhou SH Shen XQ Liu QM Fang ZF Treatment of postcatheterization femoral arteriovenous fistulas with simple prolonged bandaging. Chin Med J (Engl). (2007) 120(11):952–5. 10.1097/00029330-200706010-00002 17624260

[17] Russu E Muresan AV Kaller R Cosarca CM Arbanasi EM Arbanasi EM. Case report: gigantic arteriovenous femoral Fistula following cardiac artery catheterization. Front Surg. (2022) 9:769302. 10.3389/fsurg.2022.769302 35198595 PMC8858822

[18] Vagefi PA Kwolek CJ Wicky S Watkins MT. Congestive heart failure from traumatic arteriovenous fistula. J Am Coll Surg. (2009) 209(1):150. 10.1016/j.jamcollsurg.2008.11.021 19651078

